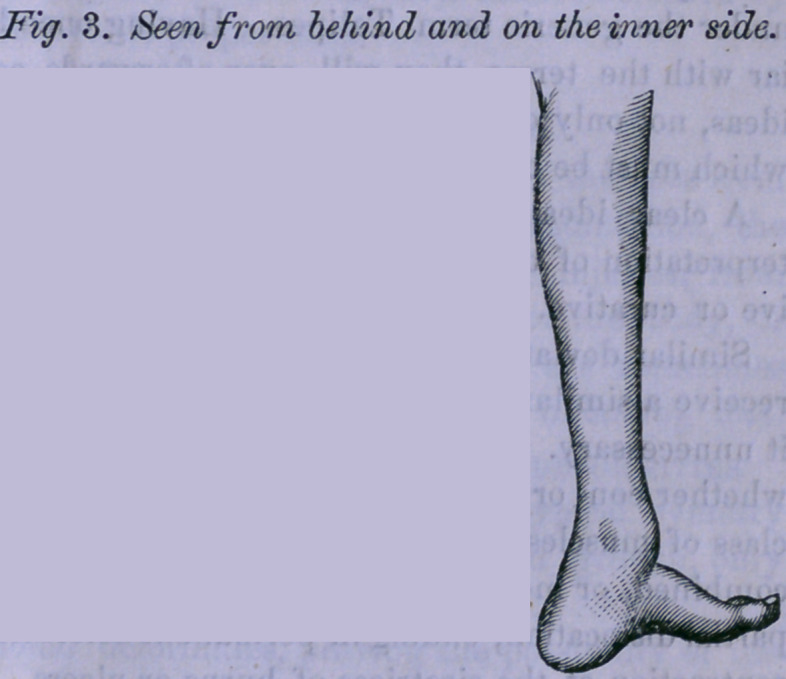# Report on Orthopedic Surgery, Made at the Annual Meeting of the Illinois State Medical Society, Convened in Chicago, May 3d, 1864

**Published:** 1864-10

**Authors:** David Prince

**Affiliations:** Jacksonville, Ill.


CHICAGO
MEDICAL JOURNAL.

VOL. XXI.] OCTOBER, 1864. [No. 10.

### ORIGINAL COMMUNICATIONS.

### REPORT ON ORTHOPEDIC SURGERY,

MADE AT THE ANNUAL MEETING OF THE ILLINOIS STATE MEDICAL SOCIETY, CONVENED IN CHICAGO, MAY 3D, 1864.

By DAVID PRINCE, M. D., Jacksonville, Ill.

NOTE.—The Verbal Analysis of the report made to the Society covered portions of the whole ground of Orthopedic Surgery. By resolution of the Society all reports were required to be furnished for publication by the 1st of July. It is impracticable with other engagements to complete the whole Report in a satisfactory manner, by that date. The portion embracing the group of deformities of the feet, known by the generic term TALIPES, is all that can appear in the Transactions for this year.

It is believed that the presentation to the profession of the latest advances in this country and in Europe, with the improvements introduced by the writer, will enable every practitioner to cure every uncomplicated case of Congenital Talipes occurring in his own practice, if undertaken during the early months of infancy.

It is also believed that most cases under fifteen years of age are capable of successful treatment, by patience, perseverance and skill.

DEFINITION AND CLASSIFICATION OF THE GENUS, SPECIES, AND VARIETIES OF TALIPES.

The term Talipes (Latin, *talus*, an ankle, and *pes*, a foot,) has come to be adopted as a generic term for what is known as club foot, reel foot, and splay foot or flat foot. The name expresses only a minor element of the deformity—the ankle in some species being not at all displaced or deformed—but this is of no great importance, since the technical signification has been agreed upon.

*Definition.*—A malposition or malformation of the foot, congenital or acquired, in which, from some deviation at the ankle joint, or in a greater or less number of tarsal or tarso-metatarsal joint, the sole of the foot fails to apply to the ground in the natural position.

Of this genus there are six species: Talipes equinus, calcaneus, varus, dorsalis, plantaris, valgus.

Of these species there are six possible secondary combinations or varieties: Talipes equino varus, equino dorsalis, equino valgus, calcaneo varus, calcaneo valgus, calcaneo plantaris.

The conception of the tertiary combinations, when once familiar, will also be simplified by classifying them thus:

Talipes equino varo dorsalis, equino valgo plantaris, calcaneo varo dorsalis, calcaneo valgo plantaris.

*Talipes equinus*, is the term applied to that position which, by long continued voluntary elevation of the heel to compensate for several inches shortening of the limb, becomes, not only habitual, but fixed, by the permanent shortening of the triceps extensor pedis, and the adaptation of the ligaments to to the habitual relations of the bones of the leg and tarsus. The habitual voluntary contraction of the triceps muscle (gastrocnemius, plantaris longus and soleus,) terminating in the tendon Achilles, becomes permanent and involuntary; after which the muscular tissue changes its character, is absorbed, or in part replaced by fat, while the white fibrous tissues investments become hypertrophied, converting the muscles into ligaments both in constitution and function. The result is a compensating deformity, and to attain the best possible compensation bringing the phalanges as nearly as possible within the vertical line of pressure, the foot comes to be more than naturally arched by the contraction of the tibialis posticus, the peroneus longus, the flexor longus digitorum, upon the back of the leg, and the adductor pollicis, the flexor brevis digitorum, the abductor minimi digiti, and the muscular accessories, with corresponding shortening of the plantar fascia under the foot. The action of the long and short flexors of the toes would curl them under the sole, as the fingers are flexed upon the palm, if they were not kept out by the weight of the body upon the phalanges.

This makes the variety T. equino dorsalis, which in the confirmed state is more common than either species unmixed. The deformity which has been described as originating in a voluntary attempt at compensation, may result from spasmodic contraction of one set of muscles, or paralysis of their antagonists.

*Talipes calcaneus.*—A deformity in which the heel comes to the ground and the anterior portion of the foot is drawn up by the disproportionate contraction of the tibialis anticus, peroneus tertius, and extensor longus digitorum. This is a deformity so rare as only to be admitted as a possibility.

*Talipes calcaneo plantaris*, is a combination, equally rare, in which the yielding is not chiefly in the triceps extensor pedis, but in the medio tarsal articulation, between the astragalus and the calcaneum behind, and the scaphoid and cuboid before, with yielding to a smaller extent, of the more anterior joints of the tarsus.

*Talipes varus.*—This is the most common of all the species, whether congenital or acquired, and consists in the inversion and rotation of the anterior half of the tarsus, which can to a slight degree be imitated by taking hold of the phalanges and metatarsus, and bending the foot in the direction in which the tibialis anticus would draw it. In making this twist, the calcaneum and astragalus will become adducted, as in the position which a child will sometimes assume in standing upon the outer edge of his foot.

Attention has been called to a better anatomy of this deformity by Mr. Barwell, in his little book, entitled "Club Foot without Division of Tendons," in which he gives the appropriate name "medio tarsal articulation" to the articulation between the calcaneum and the cuboid on the outside, and between the astragalus and the scaphoid upon the inside. "This is the centre of the twist, which in a delicate foot can almost be imitated, inward. While outward, or in even the opposite direction, there is very little capability of a twist to bring down the inner side of the sole."

In this species there is no important contraction of the triceps, through the tendon Achilles, or in other words, a corresponding elevation of the heel. The heel is tilted over as if the hand were adducting the whole foot, by taking hold of it and pulling it inward. The inner or tibial edge of the foot is turned up, and the outer or fibular side turned down, and in the worst cases, carried in toward the opposite foot, so that the outer side of the dorsum of the foot comes to the ground. The sliding of the scaphoid outward upon the astragalus makes the former bone very prominent, receiving, with the cuboid and the anterior portion of the outer and lower edge of the calcaneum, the weight of the body in standing and walking. The cuticle becomes unnaturally thickened, and between the integument and the bone, bursæ develop themselves as cushions to protect the bones from pressure in walking.

There is at first no transverse narrowing of the metatarsus and phalanges, but the pressure of walking gradually approximates the two borders of the metatarsus and phalanges; the fissure or concavity being in the plantar surface. The deformity appears to result from disproportionate contraction of the tibialis anticus, while the flexors and extensors are balanced, and tho peronei muscles paralyzed. The tibialis posticus assists in the inversion of the foot so as to make the toes point toward the opposite foot.

*Fig.* 1. *A Front View.*

This mal-position is very well illustrated by the following cuts representing the lower extremities of a gentleman 52 years of age, whose parents took him to Cincinnati when an infant, to consult the best surgeons of that city. The parents were told that nothing could be done for the child.

*Fig.* 2. *A Back View.*

It will be noticed that, in these figures, there is very little elevation of the heel through shortening of the tendon Achilles, the mal-position consisting of a remarkable twisting and doubling of the feet.

*Talipes equino varus.*—This combination is the most common variety of Talipes acquired subsequently to birth, and consists of disproportionate contraction of the triceps extensor pedis, through the tendon Achilles, elevating the heel, and making a Talipes equinus. The tibialis posticus tends to double the foot inward, while the tibialis anticus at the same time acts upon the inner edge of the foot, and rotates it, while the tibialis posticus, flexor longus digitorum, and the short flexors originating from the calcaneum, shorten the arch of the foot, making the compound expressed by the succession of terms, Talipes equino-varo-dorsalis. Walking doubles the foot still more, antero posteriorly as well as transversely, almost completely turning it upside down, giving the gait a much worse hobble than that of simple varus, and presenting a complicated deformity requiring apparatus equal to the versatility of the hand for its successful treatment.

*Talipes dorsalis.*—An unnatural elevation of the arch of the foot by a change in the medio-tarsal articulation, or the tarso-metatasal articulation, or in all combined. This condition has already been noticed in combination, in T. equino dorsalis, and T. equino varo dorsalis. It may exist as an uncombined deformity, either as a natural development, as the result of disease or injury, or as an artificial production. The shape of the foot produced by the Chinese shoe is a shortening of its length and a humping up of the instep, making a stumped appearance—a Talipes dorsalis.

It may also result from a partial dislocation, breaking up of the ligamentous fastenings on the dorsum of the foot, and permitting a shortening of the base of the tarso-metatarsal arch. This once occurred under the observation of the writer. A young man falling twenty feet, from a tree, and dislocating the tarso-metatarsal articulations of both feet. The deformity was never completely reduced, and the tarso-metatarsal joints remained permanently elevated, requiring shoes to be made according to special measurements.

*Talipes plantaris*—flat-foot.—The condition in which the sole comes to the ground in all parts; there being little or no arch. This is the natural condition in a portion of the negro race, and is often the result of want of action of the tibialis anticus and T. posticus, resulting in elongation of the plantar fascia from too great tension of it. In feeble children it comes from premature walking.

*Talipes valgas.*—The condition in which the anterior half of the foot is carried outward in the direction opposite to that of T. varus.

The tibialis anticus and tibialis posticus fail, and the peroneus longus and peroneus brevis passing behind the external malleolus, pull upon the outer side of the foot and evert it. At the same time the peroneus tertius passing down in front of the external malleolus, elevates the outer side of the foot and tilts the astragalus and calcaneum outward in the opposite direction to that taken in T. varus.

*Fig. 3. Seen from behind and on the inner side.*

The following cut illustrates this species, which is rarely met with, without complication. The figure is taken from the cast of the foot of a gentleman living in Boston. The cast is kept by Messrs. Tiemann & Co., Surgical Instrument Makers, N. Y., for the purpose of making upon it, the apparatus which aids him in walking.

It will be noticed that this is a simple Talipes valgas without any flattening of the arch of the foot to make the species plantaris. The more common development is,

*Talipes valgo plantaris.*—The condition in which the anterior half of the foot is carried outward and upward, bringing the inner side of the tarsus to the ground, while the arch of the foot is lost by the relaxation of the muscles, ligaments, and fasciæ which sustain it. As the deformity progresses, the extension or downward projection of the medio-tarsal joint, permits the metatarsis to rise altogether from the ground by the action of the peroneus tertius, leaving the weight to come altogether upon the tarsus. This extreme perversion, however, constituting a Talipes calcaneo-valgo-plantaris, is rarely attained. When existing, it must arise from the action of the extensor longus digitorum acting in concert with the peronei muscles, or more commonly, from paralysis of the opposing flexor and adductor muscles.

*Talipes calcaneo varus* is only a possible variety, resulting from disproportionate action of the tibialis anticus and tibialis posticus, the triceps extensor pedis being paralyzed, so as to prevent the long flexors to elevate the metatarsus, while the heel remains depressed.

This classification may seem unnecessary, but it is the shortest way of describing the great variety of deformities classed under the generic term Talipes. Having once become familiar with the terms, they will ever afterwards convey definite ideas, not only of the forms, but of the muscular contractions which must be concerned in producing and perpetuating them. A clear idea of these conditions will lead to a rational interpretation of the indications of treatment, whether preventive or curative.

Similar deviations from the normal form of the hand should receive a similar classification, only that their rareness makes it unnecessary. Their pathology is undoubtedly the same, whether con- or post-genital, depending upon paralysis of one class of muscles, or over-action of their antagonists, or both combined, or more rarely, some accidental injury, resulting in partial dislocation, ending in permanent deformity, or from the contraction of the cicatrices of burns or ulcers.

COMPLICATIONS.

1. The complications may be congenital or acquired absence or diminution of one or more bones, implying the impossibility of complete restoration of the form and functions of the foot, though great improvement may in some cases be effected by treatment.

2. Anchylosis of one or more joints from fractures or wounds nearly or quite helpless of benefit from subsequent treatment.

3. Anchylosis from arthritic or periosteal inflammation, in which the treatment is chiefly preventive by substituting before it is too late passive motion for absolute rest of the parts in relation to each other.

4. Contraction of cutaneous cicatrices from burns, ulcers or wounds. The treatment should be preventive; for confirmed deformities from these sources are extremely difficult to overcome.

5. Rheumatism, producing talipes, or simply attacking a talliped, requiring the abatement of the rheumatism in addition to whatever else may be done.

6. Corns and bunions requiring nice adaptation of shoes where, from the age of the patient, they cannot be cured by restoring the foot to its proper form.

7. Absence or deficiency of toes.

8. Supernumerary toes which may be cut off.

9. Deviation of the forms and directions of the toes from fractures, wounds, arthritic or periosteal inflammation, the contractions of cicatrices from burns or other injuries, from faulty shoes, from pressure of the weight of the body, or from paralysis of muscles. These deviations are sometimes incapable of remedy except by amputation of offending toes.

### CAUSES AND NATURE OF TALIPES AND ALLIED DEFORMITIES.

The nice adjustment of forces by which typical symetry is produced and maintained in all organized growth, only needs to be contemplated to secure admiration.

The exceptional deformities, proving the possibility of imperfect adjustment of these forces, or of the occurrence of accidental impediments to their exercise, only excites our attention all the more, to the nice balance observed in the ordinary working of the law of development.

In individual failures of this organic law of symetry, the question will arise as to the modes of deviation:

1. Whether from excessive nutrition, analogous to that which secures the disproportionate growth in parts which are brought to perform compensating functions, as a leg or a kidney, which, from the impairment or destruction of the opposite, is invited to perform more than its natural part.

2. From deficent nutrition direct, from the obstruction of the blood-vessels which supply it, or indirect from failure of nervous supply to the capillaries of a part, failing to open them to the supply of blood, or from accidental or artificial quietude, and ogus to that of muscles closely confined in splints and bandages while a fractured bone is uniting.

3. From accidental positions, widely varying from those which are usual and which act to produce deformities like the forces which are afterwards employed to remove them. By this means some tendons may be forced to grow too long and others permitted to become too much shortened; while the bones, which become inordinately compressed, take the shapes which the altered forces tend to give them.

4. From some observations made by Cruveilhier, this careful pathological anatomist came to the conclusion that position of the foot within the uterus was often a cause of Talipes.

As a moderate Talipes varus is the ordinary position of the foot within the uterus, this deformity can hardly be explained upon this hypothesis, but a Talipes valgus might possibly be produced by an eversion of the foot from the pull of the umbilical cord accidentally entangled around it.

Twisting and displacements and spontaneous dislocations of the knee joint, of the hip joint and of the shoulder joint can sometimes be most plausibly explained upon this supposition.

5. From the occurrence of causes which directly compress, or partially or completely cut off portions of the developing limbs. Portions of the liquor amnii unusually condensed or solidified into sheets or shreds, may produce deep fissures in parts upon which they press, or they may completely amputate the included parts. The peculiar deformities constituting the genus Talipes can hardly be explained by reference to this class of causes. Spontaneous amputations doubtless often owe their occurrence to this cause.

6. From disease directly resulting in the death of the parts affected. The writer has in his possession an aborted fœtus of four months, which exhibits gangrene of one upper extremity, including the shoulder. If this fœtus had lived, there would have been the birth of a one-armed child. Spontaneous amputations are sometimes produced by this cause, but Talipes cannot be thus explained.

7. From the union of parts of two or more individuals resulting in redundancy of number. This is the explanation of a great variety of monstrosities, but it does not apply to Talipes.

8. From an influence existing in the germinal origin of the individual, like that which determines the color of the skin, the family likeness of features and the temperament. It is thus that in same families there is a perpetuation through several generations of five fingers upon the hand and six toes upon the foot, the deficiency of a thumb or a redundant one.

Though several cases of Talipes sometimes occur in one family, and in rare cases it may be repeated in the next generation, the cases are too few to favor this explanation of its occurrence. Causes acting upon the innervation of the fœtus subsequent to the formation of the type of the individual constitute a more probable explanation.

9. From causes set in operation through physical and mental influences of the mother. As an example of physical influence: One of the common expedients for distinguishing pregnancy from enlargements within the abdomen from other causes, is to place the hand, pleviously reduced in temperature, upon the mother's abdomen, to excite a convulsive movement in the fœtus. This movement may be stimulated by the compression made by the sudden tension of the abdominal muscles induced by the cold application. On the other hand, great physical exertion and the occurrence of grave disease affecting the constitution of the circulating fluids, are followed by diminution or cessation of fœtal movements, as if from some diminution of the fitness of the blood to afford to the fœtus the highest activity of nutrition. The death of the fœtus and its expulsion is a frequent occurrence under these circumstances.

That deformities should sometimes arise from this impaired or perverted nutrition is as probable as that similar disturbances should, after birth, produce local congestions and inflammations, or convulsions and paralysis; some constitutional tendency previously induced determining the location and character of the diseased action.

Protracted mental depression, the indulgence of ungoverned anger, hate or revenge, impairing digestion, are supposed to be unfavorable to the best development of the fœtus, while the cheerful and joyous emotions are invited as most favorable.

With the shock from the sight of a repulsive object the mother feels a convulsive movement of the fœtus, followed by a diminution of habitual movements, and her attention is afterward anxiously fixed upon her own sensations and those produced in her by the fœtus. Derangement of the digestion of the mother, and the consequent impairment of the healthy and nutritive qualities of her blood, which is the source of nutriment to the fœtus, often exist for a longer or shorter period, and deformities sometimes follow; but at the birth the mother's fears are generally found to be needless, as a perfect form occupies the place of the dreaded deformity.

In the few cases that do occur there are, in exceptional instances, striking resemblances to some object seen by the mother during pregnancy; but, upon close scrutiny of the deformities, they are found to belong to classes of excessive, deficient, perverted or arrested development already referred to, from the various causes classified, and their resemblances are too few, in comparison with the whole number, to be worthy of any other explanation than that of coincidence. We all know how a striking coincidence takes more hold upon the mind than many discrepancies. The adoption, early in the civilization of all nations, of the theory of the direct production of special deformities, through the images impressed upon the mind of the mother, is probably thus best explained.

The deformities arising from spasm and paralysis are more frequent in the lower extremities from the more feeble, more easily deranged and less easily restored innervation of these parts. They are therefore more often seen in the streets, and from the awkward movements in walking they are more repulsive than deformities of the upper extremities which need not be made conspicuous in public places.

The late development and comparatively low innervation of the inferior half of the fœtus might be expected to result in the existence at birth of a greater number of deformities, produced by nervous derangements, in the inferior than in the superior half of the body. From this physiological order of development, as well as upon the hypothesis of coincidence, therefore, a mother who is shocked at the sight of a lame leg is more likely to have a child affected with Talipes than with a corresponding deformity of the hand, the deteriorating influence of the nervous impression upon the blood being more likely to result in spasmodic or paraletic afflictions of the lower than of the upper extremities of the fœtus.

*(To be continued.)*

## Figures and Tables

**Fig. 1. f1:**
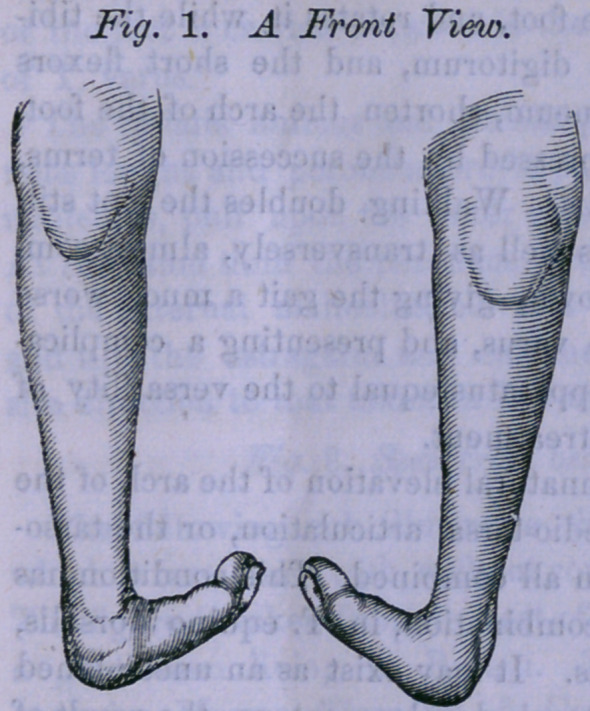


**Fig. 2. f2:**
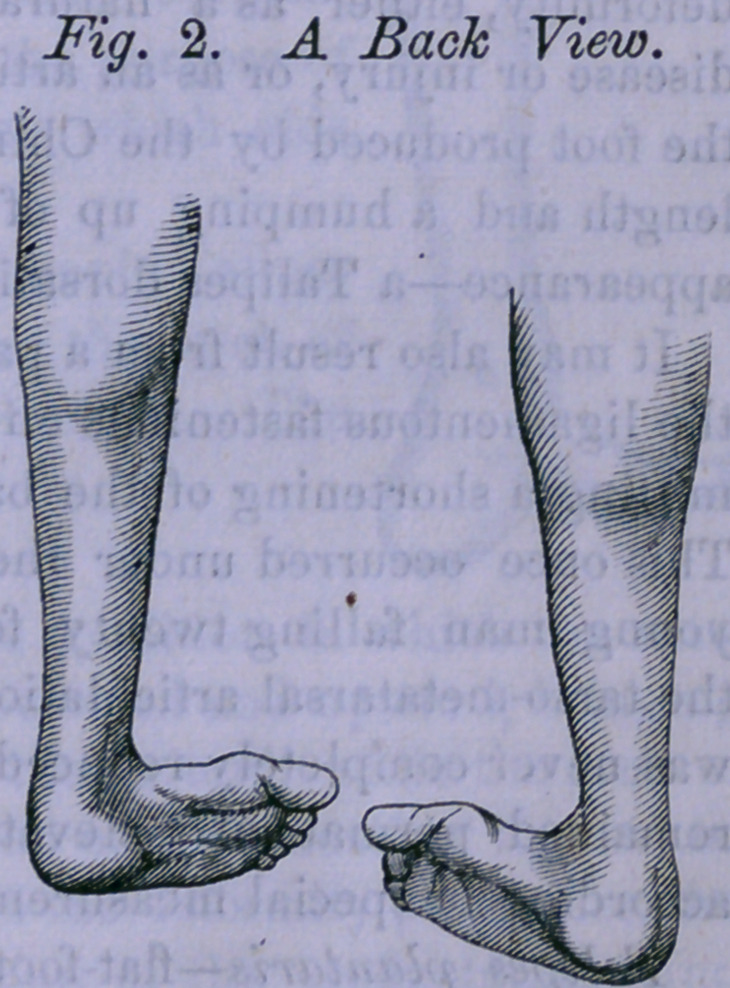


**Fig. 3. f3:**